# Comparative Assessment of Lignan Profiling and Biological Activities of *Schisandra henryi* Leaf and In Vitro PlantForm Bioreactor-Grown Culture Extracts

**DOI:** 10.3390/ph17040442

**Published:** 2024-03-29

**Authors:** Karolina Jafernik, Paweł Kubica, Michał Dziurka, Łukasz Kulinowski, Izabela Korona-Głowniak, Hosam O. Elansary, Piotr Waligórski, Krystyna Skalicka-Woźniak, Agnieszka Szopa

**Affiliations:** 1 Department of Pharmaceutical Botany, Medical College, Jagiellonian University, Medyczna 9 str., 30-688 Kraków, Poland; karolina.jafernik@doctoral.uj.edu.pl (K.J.); p.kubica@uj.edu.pl (P.K.); 2 Polish Academy of Sciences, The Franciszek Górski Institute of Plant Physiology, Niezapominajek 21 str., 30-239 Kraków, Poland; michal.dziurka@gmail.com (M.D.); p.waligorski@ifr-pan.edu.pl (P.W.); 3 Department of Natural Products Chemistry, Medical University of Lublin, Chodźki 1 str., 20-093 Lublin, Poland; lukasz.kulinowski@umlub.pl (Ł.K.); kskalicka@pharmacognosy.org (K.S.-W.); 4 Department of Pharmaceutical Microbiology, Medical University of Lublin, Chodźki 1 str., 20-093 Lublin, Poland; iza.glowniak@umlub.pl; 5 Department of Plant Production, College of Food & Agriculture Sciences, King Saud University, P.O. Box 2460, Riyadh 11451, Saudi Arabia; helansary@ksu.edu.sa

**Keywords:** PlantForm temporary immersion system, plant biotechnology, in vitro cultures, dibenzocyclooctadiene lignans, *Schisandra* lignans, antitumor activity, anti-inflammatory activity

## Abstract

This research’s scope encompassed biotechnological, phytochemical, and biological studies of *Schisandra henryi*, including investigations into its in vitro microshoot culture grown in PlantForm bioreactors (temporary immersion systems, TISs), as well as extracts from leaves of the parent plant, focusing on anti-inflammatory, antioxidant, anticancer, and antimicrobial activities. The phytochemical analysis included the isolation and quantification of 17 compounds from dibenzocyclooctadiene, aryltetralin lignans, and neolignans using centrifugal partition chromatography (CPC), HPLC-DAD, and UHPLC-MS/MS tandem mass spectrometry with triple quadrupole mass filter methods. Higher contents of compounds were found in microshoots extracts (max. 543.99 mg/100 g DW). The major compound was schisantherin B both in the extracts from microshoots and the leaves (390.16 and 361.24 mg/100 g DW, respectively). The results of the anti-inflammatory activity in terms of the inhibition of COX-1, COX-2, sPLA2, and LOX-15 enzymes indicated that PlantForm microshoot extracts showed strong activity against COX-1 and COX-2 (for 177 mg/mL the inhibition percentage was 76% and 66%, respectively). The antioxidant potential assessed using FRAP, CUPRAC, and DPPH assays showed that extracts from microshoot cultures had 5.6, 3.8, and 3.3 times higher power compared to extracts from the leaves of the parent plant, respectively. The total polyphenol content (TPC) was 4.1 times higher in extracts from the in vitro culture compared to the leaves. The antiproliferative activity against T-cell lymphoblast line Jurkat, breast adenocarcinoma cultures (MCF-7), colon adenocarcinoma (HT-29), and cervical adenocarcinoma (HeLa), showed that both extracts have considerable effects on the tested cell lines. The antimicrobial activity tested against strains of Gram-positive and Gram-negative bacteria and fungi showed the highest activity towards *H. pylori* (MIC and MBC 0.625 mg/mL).

## 1. Introduction

In plant biotechnology, among the most rapidly developing branches are micropropagation and the production of plant secondary metabolites [1,2]. Therefore, there is an extensive pursuit of new methods to enhance the efficiency of both processes. These methods often revolve around the establishment of large-scale production using bioreactor constructions [3,4,5]. Bioreactors operating as temporary immersion systems (TISs) are commonly utilized on both laboratory and commercial scales to boost the efficiency of plant propagation; for example, sugar cane (*Saccharum* spp. interspecific hybrids) [6], *Eucalyptus grandis* × *Eucalyptus urophylla* [7], *Dianthus caryophyllus* [8], and *Cannabis sativa* [9] TIS systems are also used for cultivating plant biomass to obtain pharmacologically active metabolites, such as alkaloids in *Dendrobium nobile* [10], or sesquiterpene lactones in *Thapsia garganica* [11].

One of the most versatile bioreactors used in plant biotechnology is the TIS manufactured by PlantForm (Sweden) [4,12]. Studies have indicated that it facilitates faster plant multiplication and is more effective for producing certain secondary metabolites compared to plants grown in soil [5,13,14,15,16]. The key aspect for maximizing the potential of in vitro cultures maintained in the PlantForm TIS is the experimental optimization of process parameters [4,5,15,16,17,18,19,20,21].

**Figure 1:**
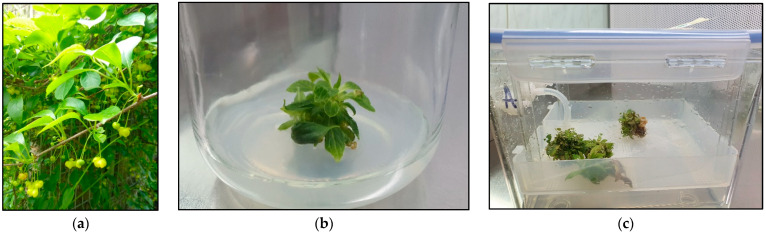
Morphological appearance of *S. henryi:* parent plant (**a**), microshoots on agar medium (**b**), microshoots grown in PlantForm bioreactor (**c**).

*Schisandra henryi* C. B. Clarke (Figure 1a) is a vine species belonging to the Schisandraceae family. It is endemic to the Yunnan Province of China, where a subtropical climate with warm summers and mild winters, along with abundant rainfall, influences its optimal growth. In European countries, this species remains relatively unknown, reflecting the limited scientific research conducted on it. To date, only two research groups from China have focused on examining the chemical composition of stem extracts and their cytotoxic activity [22,23,24,25]. Our team has conducted further studies, focusing on optimizing in vitro microshoot and callus cultures of *S. henryi* [26]. These studies have revealed that the chemical composition of *S. henryi* is similar to that of another species from the Schisandraceae family such as *Schisandra chinensis* Turcz. Baill. [27,28,29,30]. *S. chinensis* holds a strong position as a medicinal plant, as evidenced by monographs in both Asian and European pharmacopoeias [31]. The fruits of *S. chinensis* have been confirmed to exhibit various biological activities such as anticancer, hepatoprotective, anti-inflammatory, and antioxidant properties [32,33,34,35,36]. Dibenzocyclooctadiene lignans have been identified as the main compounds responsible for these therapeutic effects. This specific group of secondary metabolites is also present in *S. henryi.* Intensive research on this group of compounds continues, driven by the yet to be fully understood chemical structure and undiscovered biological activities [32,37,38,39,40,41]. Previous research has proven that dibenzocyclooctadiene lignans exhibit anticancer, hepatoprotective, antiasthmatic, antiosteoporotic, antiulcer, antioxidant, and anti-inflammatory properties [34,35,36,42,43,44,45].

The aim of this study was to develop a method for cultivating *S. henryi* microshoot cultures in PlantForm TIS bioreactors, employing tissue multiplication through in vitro methods and comparing the lignin profiles of the in vitro culture extract with that of the parent plant leaves. Tests were conducted utilizing UHPLC-MS/MS and HPLC-DAD methods for qualitative and quantitative determinations. The predominant compounds were isolated using CPC chromatography. Moreover, as part of the research, the biological activities—including antiproliferative, cytotoxic, anti-inflammatory, antibacterial, antifungal, and antioxidant potential—of extracts from multiplied microshoot biomass using a TIS were tested for the first time.

## 2. Results

### 2.1. The Microshoot Cultures Grown in PlanForm Bioreactors

The appearance of the tested microshoot cultures, whether grown on a standard solid medium or in PlantForm bioreactors, exhibited similarities (Figure 1). Microshoots cultivated in PlantForm bioreactors over a 30-day cycle displayed green leaves with no signs of tissue death. The fresh mass of microshoots in the TIS was 30.970 g. The growth index (Gi) of biomass increments of microshoots in the TIS was equal to 416.17% (Gi), which was 6.80 times higher compared to agar (basic) microshoot culture increments (Gi = 61.18%).

### 2.2. Lignan Profiling 

For the separation and determination of lignan structures, CPC fractionation followed by LC-MS/MS analysis of extracts from leaves of the parent plant was employed. Henridilactone C, pregomisin, 4-hydroxy-2-[hydroxy(3-hydroxy-4,5 dimethoxyphenyl)methyl]-3-(hydroxymethyl)butanoic acid, schisantherin G, and schisantherin B were determined as the dominant compounds in the obtained fractions (Table 1). The compounds were provisionally identified by comparing MS data (monoisotopic masses, fragmentation patterns) with those from previous studies and databases. For schisandrin B, the internal standard was also applied. The proposed identities of the main compounds of CPC fractions, their chemical structures, molecular formulas (MF), observed retention times (RT), and calculated pseudomolecular ions and fragment ions (MS/MS) are presented in Table 1. Fractions 1 and 2 contained one main component, tentatively identified as henridilactone C. The HPLC purity of the compound in both fractions was >80%. The analysis of fraction 3 showed two main components, one of which was identified as pregomisin. Fraction 4 comprised a mixture of three main components, with two putatively identified as 4-hydroxy-2-[hydroxy(3-hydroxy-4,5 dimethoxyphenyl)methyl]-3-(hydroxymethyl)butanoic acid and schisantherin G. Fractions 5–8 consisted predominantly of schisantherin B, with purities varying between 80% and 90%. 

Using the UHPLC-MS/MS method on the methanolic leaf extract, the presence of 17 lignans from dibenzocyclooctadiene lignans, aryltetralin lignans, and neolignans was identified (Table 2 and Table S1) (Figure S1). Among these, dibenzocyclooctadiene lignans were the most abundant. The highest amounts were found for schisantherin B (361.24 mg/100 g DW), schisantherin A (61.65 mg/100 g DW), and 6-O-benzylgomisin O (68.53 mg/100 g DW) (Table 2). Additionally, one lignan from the aryltetralin group, wulignan A (0.01 mg/100 g DW), was determined (Table 2). Within the neolignan group, two compounds, licarin A and B, were identified, with contents of 0.06 and 8.40 mg/100 g DW, respectively (Table 2).

In the extracts from microshoots grown in PlantForm bioreactors, the presence of the same compounds as in the leaf extracts was confirmed (Table 2). Once again, the highest contents were found for dibenzocyclooctadiene lignans: schisantherin B (390.16 mg/100 g DW), schisantherin A (73.16 mg/100 g DW), and 6-O-benzylgomisin O (63.17 mg/100 g DW) (Table 2). Only one lignan from the aryltetralin group, wulignan A1, was found, with a content of 0.01 mg/100 g DW (Table 2). Additionally, the neolignans licarin A and B were present in the microshoot extracts, with contents of 0.92 and 5.25 mg/100 g DW, respectively (Table 2).

### 2.3. Anti-Inflammatory Activity

The anti-inflammatory effects, based on the in vitro inhibition of the enzymes 15-lipoxygenase (15-LOX), phospholipase A_2_ (sPLA_2_), cyclooxygenase-1 (COX-1), and cyclooxygenase-2 (COX-2), of *S. henryi* extracts from leaves and microshoots grown in PlantForm bioreactors were investigated. The leaf and microshoot extracts of *S. henryi* demonstrated the inhibition of sPLA_2_, ranging from 19% (175 µg/mL leaf extract) to 17% (17.5 µg/mL microshoot extract), depending on the concentration of the extract. An evaluation of 15-LOX inhibition showed that both leaf and microshoot extracts from PlantForm bioreactors similarly inhibited this enzyme by 27% and 26% (at 17.5 µg/mL concentration). The most promising results were obtained for the inhibition of COX-1 and COX-2 enzymes. Microshoot extracts showed the highest activity at a concentration of 175 µg/mL, with COX-1 and COX-2 inhibition reaching 76% and 66%, respectively. The maximum inhibition of COX-1 and COX-2 using leaf extracts was 70% (at 175 µg/mL) and 34% (at 17.5 µg/mL), respectively. Extracts from in vitro cultures grown in PlantForm bioreactors showed either higher or similar anti-inflammatory activity compared to plant material (Table 3).

### 2.4. Antioxidant Potential

The microshoots grown in bioreactors showed a higher antioxidant potential than leaves, and this was consistently demonstrated across all assay methods. The observed differences in response magnitudes are characteristic of each particular assay (FC, FRAP, DPPH, or CUPRAC) and depend on the various chemical reaction mechanisms involved. The standard mixture of two compounds estimated as dominant—schisantherin A and B—showed a similar response to in vitro cultured microshoots when assayed with FC and FRAP. However, in the CUPRAC assay, the schisantherin mixture yielded a response similar to that of leaf extracts. Interestingly, the standard mixture was almost tenfold more potent than plant samples in the DPPH assay, indicating superior antioxidative power in DPPH radical quenching (Table 4).

### 2.5. Antiproliferative and Cytotoxic Activities

The antiproliferative activity was measured against cervical adenocarcinoma (HeLa), colon adenocarcinoma (HT-29), breast adenocarcinoma (MCF-7), T-cell lymphoblast-like (Jurkat), and normal human cells (HEK-293). The extracts from *S. henryi* leaves and microshoots cultured in bioreactors showed antiproliferative activity against diverse cancer cells, as shown in Table 5. 

**Figure 2:**
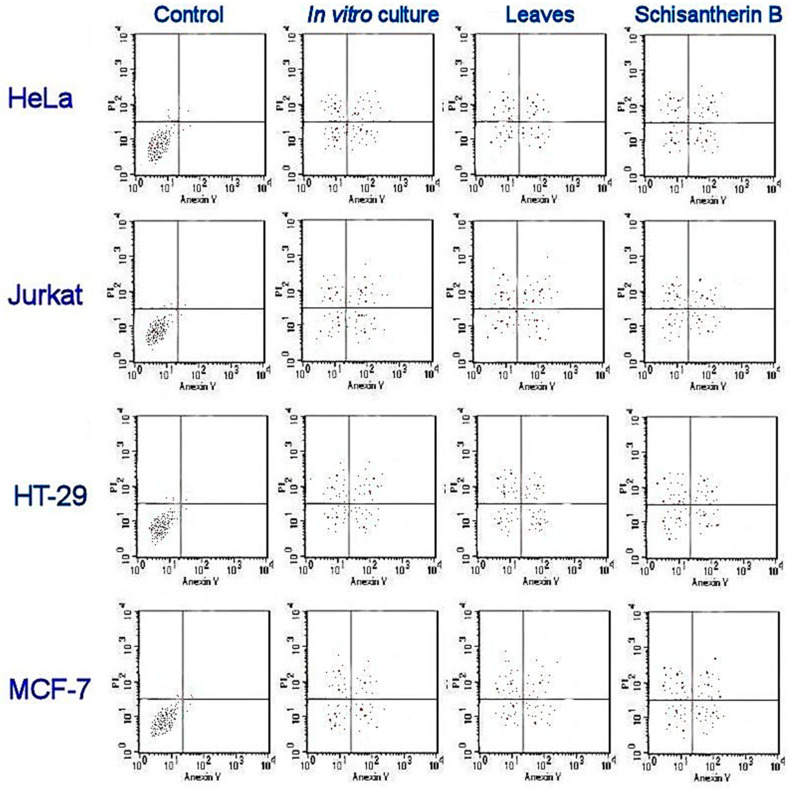
Cytotoxicity of *S. henryi* extracts from leaves and microshoots grown in PlantForm bioreactors and schisanterin B determined using flow cytometry. The control contained no extracts.

Comparatively, leaf extracts of *S. henryi* showed higher activity than microshoots from PlantForm bioreactors against different cell types. The highest activity was attributed to schisantherin B against different cancer cells. The apoptotic assay of leaf extracts of *S. henryi* and microshoots from bioreactors is shown in Figure 2, revealing necrotic cell accumulation in both the early and the late apoptotic stages.

### 2.6. Antimicrobial Activity

*S. henryi* microshoot cultures and parent plant leaf extracts were tested for their antimicrobial properties against reference bacterial and fungal strains, as shown in Table 6. 

For the *S. henryi* leaf extract, the MIC values against the tested bacterial strains ranged from 0.625 to 10 mg/mL (Table 6). Across all Gram-positive bacterial strains, the MIC values were consistently 1.25 mg/mL. However, for Gram-negative bacterial strains, the MIC values varied from 0.625 (*Helicobacter pylori*) to 10 mg/mL (*Escherichia coli* and *Pseudomonas aeruginosa*). MBC values were determined to be within the range of 0.625–10 mg/mL, with the most favorable result for MBC (0.625 mg/mL) observed against *H. pylori*. The MBC/MIC ratio, ranging from 4 to 8 for the tested bacterial strains (Table 6), indicated a bacteriostatic effect of the leaf extract.

The MIC values determined for microshoot culture extracts ranged from 0.625 to 10 mg/mL (Table 6). The Gram-positive bacterial strains (*Staphylococcus aureus* and *Staphylococcus epidermidis*) exhibited a consistent MIC value of 5 mg/mL, while for the Gram-negative bacteria, values ranged from 0.625 (*H. pylori*) to 10 mg/mL (*E. coli* and *P. aeruginosa*). The MBC of microshoot extracts ranged from 0.625 to 10 mg/mL, with the best MBC result (0.625 mg/mL) observed against the *H. pylori* reference strain. The MBC/MIC ratios, indicating a bactericidal effect of the tested extracts, were either 1 or 2 (Table 6).

For leaf extracts, the MIC values against fungal strains ranged from 5 to 10 mg/mL. The most favorable MIC results (5 mg/mL) were detected for *Candida albicans* and *Candida parapsilosis*, while the least favorable result was calculated for *Candida glabrata*, with the highest value (10 mg/mL). MFC values ranged from 10 for *C. albicans* and *C. glabrata* strains to 20 mg/mL for *C. parapsilosis*. The MFC/MIC ratio for the tested reference yeast strains ranged from 1 to 4, indicating a fungicidal effect of the tested extracts (Table 6).

The MIC value of extracts from microshoot cultures against all tested fungal strains was 10 mg/mL, while the MFC value was 20 mg/mL. With an MFC/MIC ratio of 2, these extracts demonstrated a fungicidal effect (Table 6).

## 3. Discussion

This research conducted on *S. henryi* species is pioneering as it represents the first comprehensive study on lignan profiling, both qualitatively and quantitatively. This included the utilization of modern chromatographic techniques to analyze extracts from soil-grown plant leaves and microshoot cultures multiplied in PlantForm systems. Additionally, as part of our research, the extracts were tested for their broad-spectrum biological effects, including antioxidant, anti-inflammatory, antimicrobial, and antiproliferative properties.

Qualitative analysis using UHPLC-MS/MS with a tandem mass spectrometer featuring a triple quadrupole mass filter method showed the presence of compounds from the group of dibenzocyclooctadiene and aryltetralin lignans, as well as neolignans. Extracts from microshoots grown in PlantForm bioreactors had never been analyzed for lignan content prior to this study. The quantitative analysis of *S. henryi* leaf extracts showed the same lignan profile as observed in in vitro cultures. A total of 17 lignans were identified, with a predominance of dibenzocyclooctadiene lignans, known as characteristic compounds for this species and often referred to as “Schisandra lignans” (Table S1). Moreover, the CPC isolation technique was used to obtain fractions of *S. henryi* leaf extract enriched with the main compounds (Table 1).

The study showed that extracts from microshoots grown in PlantForm bioreactors yield a higher content of lignans compared to extracts from leaves of the parent plant (Table 2). The dominant compounds in microshoot extracts were schisantherin B (390.16 mg/100 g DW), schisantherin A (73.16 mg/100 g DW), and 6-O-benzylgomisin O (63.17 mg/100 g DW). The individual compounds’ contents were 1.08 times higher, 1.18 times higher, and 1.08 times lower than their amounts in extracts from the leaves of the parent plant, respectively (Table 2). The productivity of the three dominant compounds was also calculated, determining the amount of the compound obtained per liter of medium. The values were as follows: 120.83 mg/L for schisantherin B, 22.66 mg/L for schisantherin A, and 19.56 mg/L for 6-O-benzylgomisin O. The specific productivity, which determines the average amount of the compound produced in a culture growing in 1 L of medium during 1 day, was calculated as follows: 4.03 mg/L/day for schisantherin B, 0.76 mg/L/day for schisantherin A, and 0.65 mg/L/day for 6-O-benzylgomisin O. This research has proven the high utility of in vitro cultures grown in bioreactors as an alternative source of plant raw material. This is particularly important because *S. henryi* is an endemic plant of China, with limited availability in other parts of the world. The possibility of conducting in vitro cultures in any region of the world is invaluable in this case.

The biological activity profiles of *S. henryi* extracts, as proven in this research, are exceptionally valuable. Prior knowledge on this topic was primarily derived from three studies focusing on testing isolated compounds.

Liu et al. isolated 13 lignans from *S. henryi* seeds, including epischisandrone, enshcine, schisandron, henricine, enshicine, epienshicine methyl ether, wulignan A1 and A2, schisantherin A and B, schisanhenol, deoxyschisandrin, and epiwullignan A1. Additionally, they identified two compounds from the triterpenoid group, namely, schisanhenrin and kadsuric acid. Epischisandron, epiwulignan, and wulignan A1 and A2 were found to exhibit an inhibitory effect on P-388 lymphoma cells [22,51].

Chen and his team isolated four lignans from *S. henryi* stems: isowulignan, benzoylgomisin Q, schisantherin A, and gomisin G. They tested the compounds for DNA strand cleavage and cytotoxic potential in leukemia and HeLa cell lines. Their findings revealed that gomisin G, as the sole tested compound, effectively cleaved DNA in the presence of Cu^2+^ ions at a concentration resulting in an over 50% relaxation of supercoiled DNA. In vitro cytotoxicity tests demonstrated that gomisin G induced the highest cytotoxicity in cancer cells (IC_50_ = 5.51 µg/mL). Schisantherin A and benzoylgomysin Q showed moderate cytotoxic effects on leukemic cells (IC_50_ = 55.1 and 61.2 µg/mL, respectively). Benzoylgomisin Q also showed moderate cytotoxicity (IC_50_ = 61.2 µg/mL) on HeLa cells, while schisantherin A did not affect these cells [23].

The latest and most recent study involved the isolation of 12 schinortriterpenoids (henridilactones E-O) from the leaves and stems of *S. henryi* by He et al. Subsequently, the neuroprotective activity of these compounds was assessed. The investigation focused on inducing apoptosis using corticosterone in the PC12 cell line (rat pheochromocytoma). The findings revealed that henridilactone E, H, N, and O showed the strongest neuroprotective activity by inhibiting cell apoptosis. Additionally, henridilactone O increased the number of neurites [52]. 

The research conducted in this study demonstrated a potent anti-inflammatory effect in vitro, determined by assessing the percentage of inhibition against 15-LOX, COX-1 and 2, and sPLA_2_ enzyme activity (Table 3). The tested extracts exhibited a notably high anti-inflammatory activity against COX-1 and 2. Moreover, the extract from the microshoots showed higher anti-inflammatory activity against COX-1 and 2, being 1.08 and 2 times higher, respectively, compared to the result obtained from the extract from the leaves of the parent plant.

The antioxidant potential was measured using three in vitro tests: FRAP, DPPH, and CUPRAC (Table 4). The results demonstrated that microshoot extracts exhibit a greater antioxidant activity compared to leaf extracts. Specifically, the antioxidant activities of microshoots measured using the FRAP, DPPH, and CUPRAC methods were 13.5, 19.5, and 25.93 mmol Trolox eq./100 g, respectively, which was 5.5, 3.3, and 3.8 times higher than those observed in leaf extracts. The antioxidative potential determined using the Folin–Ciocâlteu (F–C) method is often used as an indicator of polyphenol content. The assay revealed a 4.1-fold increase in total polyphenol content in in vitro cultures compared to leaf extracts (Table 4). This increase may be attributed to the common observation of phenols/polyphenols accumulating excessively in in vitro cultures as a response to artificial vegetation conditions. 

The studied extracts showed antiproliferative activity against cervical adenocarcinoma (HeLa), colon adenocarcinoma (HT-29), breast adenocarcinoma (MCF-7), and T-cell lymphoblast-like (Jurkat) cell lines, while they did not exhibit cytotoxicity towards normal human cells (HEK-293) (Table 5). Interestingly, the leaf extract displayed higher activity than the microshoot extract grown in PlantForm bioreactors.

The antimicrobial activity tests against both Gram-positive and Gram-negative bacteria proved that both extracts exhibited the highest activity against *H. pylori* bacteria. *H. pylori* is a major pathogenic agent responsible for gastric and duodenal ulcer diseases, as well as gastric carcinoma and other types of gastric and extragastric diseases. The efficacy of the widely used standard therapy for *H. pylori* infection, which involves the use of antibiotics, is at risk due to the development of drug resistance and toxicity towards the human gut microbiota. This situation urgently calls for new and selective antibacterial strategies from complementary and alternative therapies [53]. Combining phytomedicine with standard antibiotic therapy may provide an effective approach for treating *H. pylori* infections [54]. 

The current findings can be compared to previous studies conducted on agar cultures of microshoots, callus cultures, and leaves of the parent plant of *S. henryi* [26]. In that study, culture growth was optimized by testing various compositions of growth media. A quantitative analysis was conducted using the HPLC-DAD method, revealing the presence of six out of nine tested dibenzocyclooctadiene lignans in agar microshoot cultures: schisandrin, gomisin G, schisantherin A and B, deoxyschisandrin, and schisandrin C. The amounts of these compounds ranged from 0.14 mg/100 g DW to 622.59 mg/100 g DW, depending on the day of culture growth and the medium variant. The highest contents were observed for schisantherin B (622.59 mg/100 g DW), schisantherin A (143.74 mg/100 g DW), and schisandrin (61.24 mg/100 g DW). Comparing the total lignan content in extracts from microshoots grown in PlantForm bioreactors, it was found to be 1.6 times lower compared to results from agar microshoot cultures. Regarding the unit contents of individual lignans—schisantherin B and A in extracts from microshoots grown in PlantForm bioreactors—they were 1.59 and 1.96 times lower compared to agar-grown microshoots, respectively. In terms of qualitative analysis, extracts from microshoots grown in PlantForm bioreactors and agar-grown microshoots contained 17 out of 22 tested compounds and 19 out of 22 tested compounds, respectively. Regarding a previous comparative analysis of parent plant leaf extracts, leaves collected in two growing seasons (May and September) were tested [26]. The presence of six out of nine tested dibenzocyclooctadiene lignans was confirmed, matching those found in microshoot agar. The same dominant compounds were found in the leaves as in the agar microshoots—schisantherin B (48.99 mg/100 g DW), schisantherin A (4.75 mg/100 g DW), and schisandrin (8.62 mg/100 g DW). The amounts of schisantherin B and A were 7.37 and 12.97 times higher compared to the results obtained in leaf extracts in this study, respectively. The total lignan content tested in the previous study was 66.74 mg/100 g DW, which was 7.65 times lower than in this study. Additionally, the current study did not detect the presence of schisandrin, although this outcome is considered more reliable due to the identification performed using HPLC-MS/MS.

The obtained results should also be compared with another species of the *Schisandra* genus—*S. chinensis*, which has monographs in many pharmacopoeias around the world; and *Sarcandra rubriflora*, which, although less popular, also possesses significant medicinal potential.

Szopa et al. conducted research on *S. chinensis* microshoots grown in PlantForm bioreactors in 30- and 60-day breeding cycles [55]. Higher results were achieved in the 30-day breeding cycle. The presence of 14 lignans was confirmed, with the maximum total lignan content reaching 546.98 mg/100 g DW, which is comparable to the results obtained from extracts of *S. henryi* microshoots grown in PlantForm bioreactors. Schisantherin B (9.85 mg/100 g DW) and schisantherin A (2.97 mg/100 g DW) were identified in the extracts from *S. chinensis* microshoots. The content of those compounds was 39.61 and 24.63 times lower, respectively, than in the results obtained from extracts of *S. henryi* in vitro microshoots.

Szopa et al. also conducted research on the species *S. rubriflora*, focusing on stimulating lignan production using elicitors in microshoots grown in PlantForm bioreactors [56]. Elicitors such as chitosan, yeast extract, ethephon, and methyl jasmonate were employed. The highest total content of *S. rubriflora* microshoots grown in PlantForm bioreactors was achieved with the addition of methyl jasmonate, reaching 152.20 mg/100 g DW. This result was 3.56 times lower than the results obtained from *S. henryi* microshoots grown in PlantForm bioreactors. The highest contents of individual lignans in extracts from *S. rubriflora* microshoots were observed for gomisin A (41.01 mg/100 g DW), schisandrin (37.6 mg/100 g DW), and deoxyschisandrin (35 mg/100 g DW), which were 205.05, 107.42, and 107.42 times higher compared to results *from S. henryi* microshoot extracts, respectively. When comparing the lignans with the highest content obtained in extracts from *S. henryi* microshoots—schisantherin B and A—they were 84.81 and 812.88 times higher, respectively, than the results from *S. rubriflora* microshoots. 

The biological activity of extracts from microshoots grown in PlantForm bioreactors and leaves of the parent plant was tested for the first time, making it challenging to perform a comparative analysis of the species.

When comparing the obtained results regarding biological activity, the species *S. rubriflora* should be included in the discussion. Szopa et al. examined extracts from *S. rubriflora* leaves and microshoot cultures [57]. Their findings revealed that the extract from *S. rubriflora* leaves exhibited the highest antioxidant potential, as assessed using the FRAP, DPPH, and CUPRAC methods. In our research, extracts from *S. henryi* microshoots grown in PlantForm bioreactors showed higher antioxidant potential. Studies on the anti-inflammatory activity of leaf and in vitro microshoot extracts of *S. rubriflora* and *S. chinensis* showed that extracts from *S*. *rubriflora* leaves (from both female and male species) displayed significant inhibitory activity against 15-LOX [45]. Comparing these results, extracts from *S. henryi* leaves were 1.56 times lower in inhibitory activity than extracts from female leaves of *S. rubriflora*. A previously tested *S. chinensis* leaf extract showed inhibitory activity against 15-LOX at a concentration of 17.5 µg/mL, and for COX-1 and 2 and sPLA_2_ at a concentration of 175 µg/mL, showing reductions of 28%, 51%, 53%, and 25%, respectively [45]. These values were 1.07 times higher, 1.37 times lower, 1.61 times higher, and 1.31 times higher, respectively, compared to *S. henryi* leaf extracts. 

Contemporary research into the popularization of the in vitro cultivation of medicinal plants often focuses not only on developing their micropropagation protocols but also on examining the production of secondary metabolites in the biomass cultivated using this method. This research is extremely valuable for utilizing such biomass to obtain active secondary metabolites with valuable medicinal properties. The results of these tests are often compared to plant material harvested from soil, and it is preferable for them to be associated with the mother/parent plants [45,57,58]. Jakabfi-Csepreg et al. [59] analyzed the antimicrobial activity of an aqueous extract from the leaves of *Lathyrus tuberosus*, a species used in European and Asian folk medicine. The aqueous extract of *L. tuberosus* leaves underwent testing for antioxidant potential, antimicrobial, and cytotoxic activity. The leaf extract, analyzed quantitatively, showed a high content of polyphenolic compounds and showed high antioxidant potential based on the DPPH test, which indicated that the ethanol extract (IC_50_ 1.42 mg/mL and 1.80 mg/mL) had a significant antioxidant effect, similar to the *S. henryi* leaf extract. Antimicrobial activity against the Gram (–) and Gram (+) bacteria of *L. tuberosus* was also evaluated. It was observed that the ethanol extract was active against *Bacillus subtilis* and *Streptococcus pyogenes* bacteria out of the four strains tested. The *S. henryi* extract showed antibacterial potential against *H. pylori*. Kowalczyk et al. [60] conducted research on the biological activity of extracts from *Plectranthus scutellarioides*. In the initial stage of this research, an in vitro culture was established from the above-ground parts of the plant and roots. Tests were carried out to assess the potential to induce apoptosis in breast (MCF-7) and lung (A549) cancer cells. It was found that both extracts from microshoot and root cultures had cytotoxic effects on cancer cells, similar to the tests performed under this study of *S. henryi* microshoot culture extract. Mantovska et al. [58] focused on the endemic species *Stachys scardica*. In vitro microshoot cultures were established, and *S. scardica* extracts were examined for phytochemical analysis and biological activities. The antioxidant potential was assessed using DPPH and FRAP tests. The results obtained for extracts from in vitro, ex vitro, and in situ cultures of *S. scardica* were 62%, 90%, and 77%, respectively, at a concentration of 60 µg/mL, indicating a significant difference compared to the results obtained in the study where the *S. henryi* microshoot cultures showed almost twice the antioxidant activity in the DPPH test compared to the results obtained from extracts from the leaves of the parent plant. Tests on the anti-inflammatory activity of *S. scardica* extracts were also conducted using a hemolytic test, which examined the effect on the complement system via the classical route. It was found that the activity of extracts from in vitro cultures of *S. scardica* was almost twice as low (57%) compared to the results obtained from extracts of plants in situ and ex vitro, whose anti-inflammatory activity was 96% in both cases, at a concentration of 2000 µg/mL. The reported results also diverged when comparing the anti-inflammatory activity of *S. henryi* extracts, where microshoot cultures showed a significant difference in the percentage of inhibition of COX-1 and 2 enzymes compared to the *S. henryi* leaf extract. Tests were also performed on the antimicrobial activity against skin strains of *S. epidermidis*, *Cutibacterium acnes* (isolated), *Bacillus cereus*, *E. coli*, *C. albicans*, and *Malassezia furfur* using a disc diffusion test. Ex vitro extracts of *S. scardica* exhibited strong inhibitory activity against *S. epidermidis* and *C. acnes* (100% inhibition), and moderate activity against *B. cereus*. Additionally, the antimicrobial activity of ex vivo plant extracts was three times higher than in extracts from in vitro cultures. Comparing the results obtained with *S. henryi* extracts, the antimicrobial activity was comparable.

**Figure 3:**
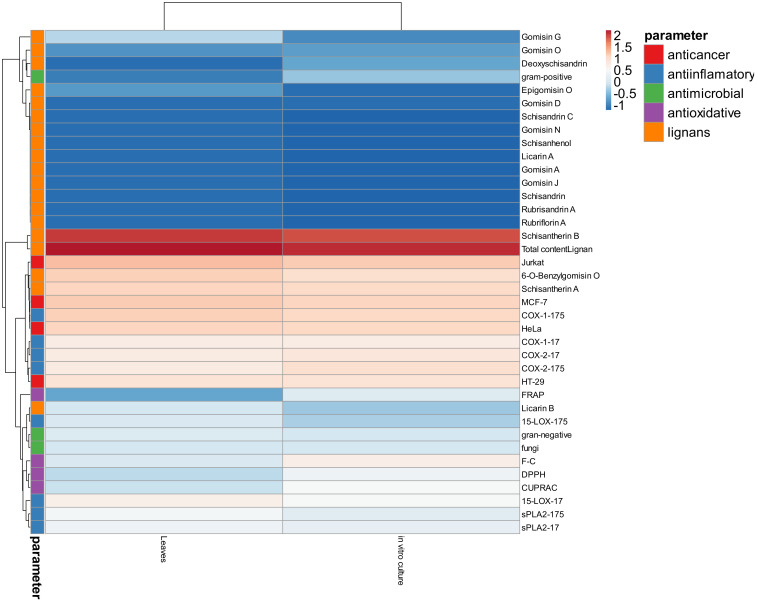
Heat map of analyzed parameters for leaves and in vitro culture biomass. Color intensity represents the relative value of a particular parameter. Red shades represent accumulation, whereas blue shades represent parameter decrease. Original values are ln(x)-transformed. Columns are centered; unit variance scaling is applied to columns. Rows are clustered using Euclidean distance and average linkage. Columns are clustered using correlation distance and average linkage.

This work presents a multidisciplinary approach to elucidate biochemical differences between plant material of a natural origin and an in vitro bioreactor culture. It gives some insights into the extracts’ anticancer, antimicrobial, or antioxidative properties. The complex matrix of mutual interactions of profiled parameters is presented in Figure 3. Using a cluster analysis, the presented heat map sums up the observed differences. Clearly, the leaf material cluster is separated from in vitro material. More interestingly, the assayed parameters are clustered in two main groups. Most accumulated lignans and the total lignan sum are closely related to anticancer and anti-inflammatory properties, the second of which is connected with inhibiting COX enzymes. Somewhat more related are the antioxidative properties and antimicrobial properties against fungi and Gram-negative bacteria. This cluster also neighbors parameters representing anti-inflammatory activity against LOX and sPLA enzymes. 

An interesting observation is that Gram-positive inhibition is more likely connected with the least abundant lignans, which suggests that this lignan cluster should be more intensively tested in this context. Noteworthy is the observation that bioreactor culture has a positive influence on gomisins and deoxyshisandrin accumulation, thus slightly stimulating anti-Gram-positive properties.

In conclusion, the research presented in this study on an *S. henryi* in vitro culture maintained in PlantForm bioreactors, which could serve as a cost-effective alternative to the scarce raw material endemic to the Yunnan Province in China, is highly intriguing and promising. The development of a phytochemical profile for both the in vitro cultures and extracts from the leaves of the parent plant, along with their comparison and a wide range of biological tests conducted, indicate the significant medicinal potential of this species. This potential may be linked to traditional Chinese medicine indications as well as the high medicinal value of the main species from the Schisandraceae family, which is rich in specific lignans, particularly the pharmacopoeial species *S. chinensis.*

## 4. Materials and Methods

### 4.1. Parent Plant Material

Material from the parent plant was obtained through collaboration with the company “Clematis” [61], which provided leaves and stems from male *S. henryi* specimens to initiate in vitro cultures. The plants were identified by Dr. Szczepan Marczyński (owner of Clematis) and Prof. Agnieszka Szopa. The leaves were harvested in May 2018 and dried in a dryer at 25 °C.

### 4.2. In Vitro Microshoot Cultures

Microshoot cultures of *S. henryi* were grown and multiplied for experiments on agar-solidified (7 g/L) plant culture medium following the Murashige and Skoog (MS) [62] protocol, supplemented with plant growth regulators: 2 mg/L indole-3-butyric acid (IBA), and 0.5 mg/L 6-benzyladenine (BA). These regulators, as shown in previous studies, are beneficial for tissue growth and the accumulation of secondary metabolites [26].

### 4.3. Experimental Microshoot Cultures Grown in PlantForm Bioreactors 

The PlantForm bioreactor, utilizing a temporary immersion system (TIS) [12] (Plant Form MWb & AJS, Hjärup, Sweden), was inoculated with 6 g of microshoots per 500 mL of liquid MS medium (6/500 *w*/*v*) supplemented with 2 mg/L IBA, 0.5 mg/L BA, and 30 g/L sucrose (Sigma-Aldrich^®^, Saint Louis, MI, USA). The cultures were grown under constant artificial light (Philips white fluorescent lamps, 90 ± 2 μmol m^−2^ s^−1^) at a temperature of 24 ± 2 °C. The cultures were maintained in the bioreactors for 30-day growth periods. The resulting biomass was evaluated in terms of parameters such as fresh mass (FW), dry mass (DW), and growth index (Gi). The Gi was calculated based on the weight of dry tissue using the formula: Gi = (DW_1_ − DW_0_)/DW_0_, where DW_0_ is the weight of the inoculum and DW_1_ is the final weight of tissue after the culture growth period [63]. Subsequently, the obtained biomass was lyophilized (Labconco Corporation, Kansas City, MO, USA), pulverized, and subjected to extraction and phytochemical analysis. The experiments in the bioreactor were conducted in three repetitions (*n* = 3).

### 4.4. Extraction 

Methanol extracts were prepared from leaves of the parent plant and microshoot culture cultivated in the TIS. Samples (0.2 g, 3 replicates) were extracted with 2 mL of methanol (HPLC grade, Merck, Darmstadt, Germany). The extraction process was carried out twice in an ultrasonic bath (Sonic 2, POLSONIC Palczyński Sp. J., Warsaw, Poland) for 30 min each time. The obtained extracts were then centrifuged for 5 min at 4000 rpm (MPW-223E, MPW, Warsaw, Poland). The collected extracts were filtered using Millex^®^ GP, 0.22 µm, Filter Unit (Millipore, Bedford, MA, USA).

### 4.5. Chromatographic Separation with CPC and Identification with HPLC-DAD and UHPLC-MS/MS 

To determine the suitable two-phase solvent system for CPC separation, the partition coefficient (*K_i_*) values of extract components were evaluated through shake-flask experiments using various combinations of *n*-hexane, ethyl acetate, methanol, and water. The optimal partition coefficient values for the components should fall within or close to the preferred “sweet spot” range (0.4 < *K_i_* < 2.5, preferably close to 1). These values ensure optimal resolution, productivity, and solvent consumption in CPC [64]. For each experiment, 5 mg of the extract was mixed with equal volumes (2 mL) of the upper and lower phases of pre-equilibrated solvent systems and vigorously shaken for 2 h. Subsequently, exactly 1 mL of the upper and 1 mL of the lower phase of each solvent system were evaporated to dryness. The resulting residues were dissolved in 1 mL of methanol and analyzed using HPLC. The partition coefficients of each component (*K_i_*) were then calculated using the following equation:

$$K_{i}=\frac{C_{i}^{UP}}{C_{i}^{LP}}$$

where $C_{i}^{UP}$ and $C_{i}^{LP}$ represent the concentrations of solute *i* in the upper and lower phases, respectively.

The selected two-phase solvent system consisted of *n*-hexane, ethyl acetate, methanol, and water in a volumetric ratio of 6/4/6/4. To prepare the appropriate system composition, the solvents were mixed in the required volume proportions, vigorously shaken, and equilibrated for at least 2 h at room temperature. The upper and lower phases were separated using a separatory funnel. These phases were then transferred to individual glass bottles and degassed in an ultrasonic bath before use.

CPC separation was conducted using a centrifugal partition chromatography (CPC) unit (CPC250 Classic, Gilson, Paris, France) with a column volume of 250 mL. The unit was connected to an AP-MOD-250 mobile phase pump (Gilson, France), a TOY18DAD PDA scanning UV detector (Ecom, Prague, Czech Republic), and a fraction collector FC 204 (Gilson, France). All CPC analyses were performed in the descending mode, where the upper phase of the two-phase solvent system served as the stationary phase and the lower phase as the mobile phase. The maximum achievable rotation speed was 3000 rpm, and the entire column could operate at a maximum pressure drop of 100 bar. The flow rate was set at 10 mL/min. Once hydrodynamic equilibrium was achieved (indicated by the mobile phase flowing from the column), a 10 mL feed sample was introduced through a manual injection valve. The effluent from the column was monitored at 254 nm. Subsequently, all 1 min fractions were evaporated to dryness, dissolved in 1 mL of methanol, and analyzed using HPLC-DAD.

The UHPLC-DAD analysis of the extract, separated fractions, and isolated compounds was performed using Shimadzu HPLC equipment (Shimadzu, Tokyo, Japan) coupled with an automatic degasser (DGU-20A 3R), a quaternary pump (LC-20AD), an auto-sampler (SIL-20A HT), a DAD detector (SPD-M20A), and a Gemini 5 μm, NX-C18, 110 Å, 250 × 4.6 mm column. A gradient of water (A) and methanol (B) was used as follows: 0 min, 50% B; 5 min, 60% B; 20 min, 80% B; 25 min, 100% B; 26 min, 50% B; and 35 min, 50% B. The flow rate was 1 mL/min, the column temperature was maintained at 25 °C, the injection volume was 10 μL, and the detection wavelength was set at 230 nm.

HPLC-MS/MS analysis was performed using an Agilent 1200 HPLC system (Agilent Technologies, Santa Clara, CA, USA) equipped with an auto-sampler (G1329A), a degasser (G1379B), a binary pump (G1312C), a column oven (G1316B), and a DAD detector (G1315B), along with a QTOF LC–MS (Agilent 6210). The experiments were carried out on a Phenomenex Gemini C18 column (2 × 100 mm, 3 μm). A solvent mixture of 0.1% formic acid in water (A) and 0.1% formic acid in acetonitrile (B) at a flow rate of 0.2 mL/min was used. The gradient program employed was as follows: 0–45 min, 5–60% B; 46–55 min, 95% B. The injection volume was 10 μL. Detection was carried out in both negative and positive electrospray ionization modes, with MS scanning conducted in the range of m/z 50–1700. The ion source parameters were set as follows: drying gas (N2) flow rate, 10 L/min; drying gas (N2) temperature, 275 °C; sheath gas (N2) flow rate, 12 L/min; sheath gas (N2) temperature, 325 °C; nebulizer pressure, 35 psi; capillary voltage, 4000 V; nozzle voltage, 1000 V; fragmentor voltage, 140 V; skimmer voltage, 65 V; octapole RF peak voltage, 750 V. The MS/MS analyses were conducted with collision dissociation energies set at 10 and 30 V. Data processing was performed using MassHunter v. B08.00 (Agilent Technologies, Santa Clara, CA, USA).

### 4.6. Chromatographic Profiling with UHPLC-MS/MS

Quantitative analysis of lignans was performed using a UHPLC-MS/MS tandem mass spectrometer with a triple quadrupole mass filter (QQQ) (Agilent 6410 LC/MS) connected to an ultra-high-performance chromatograph (Agilent 1260), as detailed previously [45]. The separation was achieved on a Kinetex C18 analytical column (150 × 4.6 mm, 2.6 μm) using a gradient of 50% methanol (A) vs. 100% methanol (B), both with 0.1% formic acid (ramped from 20% to 65% B over 22 min at a flow rate of 0.5 mL/min, a column temperature of 60 °C, and an injection volume of 2 μL). The analyses were carried out in positive ionization mode (+ESI) using selected ion transitions (MRM mode). Identification and quantification were performed by comparing lignans with pure standards obtained from ChemFaces Biochemical Co., Ltd. (Wuhan, China). The quantitated lignans included wulignan A1, rubrisandrin A, interiotherin C (rubriflorin A), schisandrin A, gomisin D, gomisin J, gomisin, gomisin G, licarin B, epigomisin O, gomisin O, meso-dihydroguaiaretic acid, schisantherin A, schisantherin B, licarin A, schisanhenol, deoxyschisandrin, fragransin A, pregomisin, gomisin N, 6-O-benzylgomisin O, and schisandrin C; and for leaf extracts this included schisandrin, gomisin A, gomisin G, schisantherin A, schisantherin B, schisanhenol, deoxyschisandrin, γ-schisandrin, and schisandrin C (Figure S1). Further technical details are provided by Szopa et al. [45].

### 4.7. Anti-Inflammatory Activity

Methanol extracts from the leaves and microshoots of *S. henryi* were assessed for their anti-inflammatory activity. The plant extracts were diluted serially in methanol. The tests focused on inhibiting the enzymes 15-lipoxygenase (15-LOX), phospholipase A_2_ (sPLA_2_), cyclooxygenase-1 (COX-1), and cyclooxygenase-2 (COX-2) in vitro. Measurements were performed in a 96-well plate using a Synergy II reader (Biotek, Winooski, VT, USA). Each sample underwent triplicate testing, which included assessing 100% enzyme activity, positive inhibitor control, and the test extracts.

For the inhibition activity of 15-LOX, samples were tested using an assay kit (760700, Cayman Chem. Co., Ann Arbor, MI, USA) following the manufacturer’s instructions, with arachidonic acid at 0.91 mM as the substrate and nordihydroguaiaretic acid (NDGA) at 100 µM as the positive control. Measurements were taken at 490 nm. 

To assess the inhibition of COX-1 and COX-2, samples were tested using a COX-1 (ovine) and COX-2 (human) inhibitor assay kit (701050, Cayman Chem. Co., MI, USA) as per the manufacturer’s instructions, with arachidonic acid at 1.1 mM as the substrate and ibuprofen at 10 μM as the positive control. The kit measured the COX peroxidase component, and the reaction was kinetically monitored for 5 min in a 96-well plate at 590 nm.

The inhibition of sPLA_2_ activity was assessed using an assay kit (10004883, Cayman Chem. Co., MI, USA) following the manufacturer’s instructions. The substrate used was diheptanoyl thio-PC at 1.44 mM, with thioetheramide-PC at 100 µM serving as the positive control. The reaction was kinetically monitored in a 96-well plate format at 420 nm. 

The percentage of inhibition was calculated using the following equation:

%Inh = [(IA-Inhibitor)\IA] × 100

where %Inh represents the percentage of inhibition, IA denotes 100% enzymatic activity (no inhibitor), and IA-Inhibitor signifies the enzyme activity with an added inhibitor. 

### 4.8. Antioxidant Potential and Assay of Total Phenolic Content 

Approximately 0.05 g of dry weight (DW) precisely weighed homogenized samples were extracted using 1 mL of methanol for 10 min at 30 Hz (MM400, Retch, Haan, Germany). The samples were then centrifuged (3 min, 15 °C, 33,000× *g*, 32R, Hettich, Tuttlingen, Germany), and the supernatant was collected for further analyses. All analyses were conducted in a 96-well plate format as described by Szopa et al. [14]. The Synergy II reader (Biotek, Winooski, VT, USA) was used. All chemicals were of analytical grade supplied by Sigma-Aldrich (Poznań, Poland). The value of antioxidant activity is presented as Trolox equivalent (nmol of Trolox per mg of DW of the sample).

The ferric reducing ability of plant extracts was determined using the FRAP (ferric reducing ability of plasma) method described by Benzie and Strain [65]. 

Free radical-scavenging activity measurements were performed using the stable radical DPPH [66]. 

The CUPRAC method [67] was adopted as detailed by Biesaga-Kościelniak et al. [68] and Dziurka et al. [69]. Technical details are provided by Szopa et al. [14].

The Folin–Ciocaletu (F–C) assay was conducted according to the method of Singelton et al. [70] with modifications reported by Bach et al. [71]. 

Technical details for these assays are outlined by Szopa et al. [45]. 

### 4.9. Antiproliferative Activitiy 

The antiproliferative and cytotoxic activities of *S. henryi* leaf and microshoot extracts were examined against Jurkat, MCF-7, HT-29, HeLa, and HEK-293 (normal human cells) [72,73,74]. The dried extracts were solubilized in DMSO (1%). Cancer cells were cultured in standard media (MEM) containing 10% FBS, 0.1 mM nonessential amino acids, 17.8 mM NaHCO_3_, and 1 mM sodium pyruvate in 75 cm^2^ flasks. Cells were seeded in microplates at 4 × 10^−4^ cells per µL in 270 µL medium and incubated for 48 h at 37 °C with 5% CO_2_. Subsequently, the extracts were added to achieve final concentrations of 50, 100, 200, 300, and 400 µg/mL. After washing with PBS, 12 mM of MTT (dissolved in PBS) was added to the medium, followed by isopropanol (0.04 M HCl) to each well, and left for 40 min. Negative control (untreated), positive standards (vinblastine sulfate and taxol), and schisantherin B were included. The percentage of inhibition activity (IAA) was calculated using the absorbance measured at a wavelength of 570 nm, employing the following equation:

$$IAA=\frac{{\left({AB}_{570nm}\right)}_{C}-{\left({AB}_{570nm}\right)}_{s}}{{\left({AB}_{570nm}\right)}_{C}}\times 100$$

where *AB* represents absorbance, ${\left({AB}_{570nm}\right)}_{C}$ and ${\left({AB}_{570nm}\right)}_{s}$ represent the absorbance at 570 nm of control and sample, respectively.

To determine the IC_50_, the percentage of viable cells was plotted against extract concentration in µg/mL using flow cytometry (FAC Scan, USA) [72,74,75].

### 4.10. Antimicrobal Activity

The extracts from *S. henryi* leaves and bioreactor microshoot cultures underwent screening for antibacterial and antifungal activities using the microdilution broth method. Mueller–Hinton broth and Mueller–Hinton broth with 5% lysed horse blood were utilized for the growth of nonfastidious and fastidious bacteria, respectively, while RPMI (Roswell Park Memorial Institute Medium) with MOPS (3-(N-morpholino)propanesulfonic acid) was employed for fungal growth, as described elsewhere [76,77]. The quantitative method of minimal inhibitory concentration (MIC) and minimal bactericidal/fungicidal concentration (MBC/MFC) determination was conducted for a diverse panel of reference strains from the American Type Culture Collection (ATCC). This panel included Gram-negative bacteria (*E. coli* ATCC 25922, *P. aeruginosa* ATCC 9027, *H. pylori* ATCC 43504), Gram-positive bacteria (*S. aureus* ATCC 25923, *S. aureus* ATCC 43300, *S. epidermidis* ATCC 12228), and fungi (*C. albicans* ATCC 10231, *C. glabrata* ATCC 90030, *C. parapsilosis* ATCC 22019, *Aspergillus niger* ATCC 16404). Each experiment was conducted in triplicate, and representative data were presented. Vancomycin, ciprofloxacin/ofloxacin, and nystatin/amphotericin B served as the standard reagents for Gram-positive, Gram-negative, and fungal reference strains, respectively.

### 4.11. Statistical Analysis

Quantitative results from chromatographic assays are expressed in mg/100 g DW as the mean ± SD (standard deviation) of three (*n* = 3) triplicate experiments. 

Differences in particular measured parameters between the leaf and in vitro samples were estimated using a Student’s *t*-test at a significance level of 0.05. The calculations were performed using Statistica 13.1 software.

The heat map was created with the use of an online tool (http://biit.cs.ut.ee/clustvis/ accessed on 29 April 2022) according to [54].

## Figures and Tables

**Table 1:** Lignans of *S. henryi* leaf extract and their mixtures separated using CPC and identified based on LC-MS/MS method.

No.	Proposed Identification	Chemical Structure	Exp. (*m/z*)	Calc. (*m/z*)	RT [min]	MF	MS/MS (+)	References
1	Henridilactone C	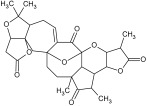	527.3455 [M + H]^+^	527.22756	17.20	C_29_H_34_O_9_	275.1773	[46]
2	Henridilactone C		527.3426 [M + H]^+^	527.22756	17.28	C_29_H_34_O_9_	275.1745	[46]
3	Unidentified		309.2130		30.6		291.2030, 273.1955, 251.3999, 275.1822	
	Pregomisin	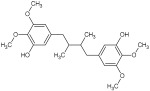	391.2208 [M + H]^+^	391.21206	32.2	C_22_H_30_O_6_	359.1998, 337.9215, 237.1592, 205.1227, 167.0729	[47]
4	4-Hydroxy-2-[hydroxy(3 hydroxy-4,5 dimethoxyphenyl)methyl]-3 (hydroxymethyl)butanoic acid	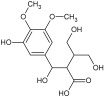	317.2567 [M + H]^+^	317.24806	30.9	C_21_H_32_O_2_	300.2302, 299.2393, 219.1318	[48,49]
	Schisantherin G	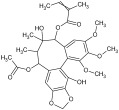	559.2032 [M + H]^+^	559.21794	36.2	C_29_H_34_O_11_	438.1609, 416.2209, 372.1746, 237.8928	[47]
	Unidentified		611.4284		39.2		550.5979, 508.2968, 317.2182	
5	Schisantherin B	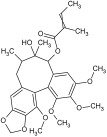	537.2235 [M + Na]^+^	537.2095	35.9	C_28_H_34_O_9_	438.1570, 416.1903, 372.1536, 342.1123	[47,50]
6	Schisantherin B		537.2245 [M + Na]^+^	537.2095	36.0	C_28_H_34_O_9_	438.1664, 416.1982, 372.1624	[47,50]
7	Schisantherin B		537.2195 [M + Na]^+^	537.2095	36.0	C_28_H_34_O_9_		[47,50]
8	Schisantherin B		537.2255 [M + Na]^+^	537.2095	36.1	C_28_H_34_O_9_	438.1748, 416.1893, 372.1726	[47,50]

**Table 2:** Contents (mg/100 DW ± SD) of lignans in *S. henryi* extracts from leaves and microshoots grown in PlantForm bioreactors.

Lignans	Leaves of Parent Plant	Microshoot In Vitro Culture Grown in PlantForm Bioreactors
Wulignan A1	0.01 ± 0.00	0.01 ± 0.00
Rubrisandrin A	0.08 * ± 0.00	0.04 * ± 0.00
Rubriflorin A	0.02 * ± 0.00	nd *^‡^
Schisandrin	nd *	0.35 * ± 0.20
Gomisin D	0.85 * ± 0.12	1.15 * ± 0.31
Gomisin J	0.40 * ± 0.09	0.23 * ± 0.19
Gomisin A	0.02 * ± 0.00	0.20 * ± 0.17
Gomisin G	5.62 * ± 1.98	1.90 * ± 0.12
Licarin B	8.40 * ± 2.56	5.25 * ± 0.89
Epigomisin O	1.97 ± 1.05	1.21 ± 0.15
Gomisin O	1.76 ± 1.00	2.54 ± 0.21
Schisantherin A	61.65 * ± 4.05	73.16 * ± 4.31
Schisantherin B	361.24 * ± 13.06	390.16 * ± 12.61
Licarin A	0.06 * ± 0.00	0.92 * ± 0.14
Schisanhenol	0.02 ± 0.00	0.03 ± 0.00
Deoxyschisandrin	0.03 * ± 0.00	3.02 * ± 0.23
Gomisin N	0.17 * ± 0.05	0.62 * ± 0.16
6-O-Benzylgomisin O	68.53 ± 3.17	63.17 ± 3.12
Schisandrin C	0.01 ± 0.00	0.03 ± 0.00
Total content	510.84 * ± 27.13	543.99 * ± 22.81

^‡^ not detected; asterisk (*) denotes statistical significance within the row for measured parameter between leaf and in vitro material according to Student *t*-test (at α < 0.05).

**Table 3:** Anti-inflammatory activity estimated based on the in vitro inhibition (% Inh. ± SD) of 15-LOX, COX-1, COX-2, and sPLA_2_ enzymes of *S. henryi* extracts from leaves and microshoots grown in PlantForm bioreactors.

Extracts		In Vitro Enzymes Inhibition			
		15-LOX	COX-1	COX-2	sPLA_2_
	Concentration μg/mL	% Inh ± SD			
Leaves of parent plant	175.0	9 ± 0.6	70 ± 7.7	33 ± 3.7	19 * ± 0.8
	17.5	27 ± 1.9	31 * ± 3.4	34 * ± 3.8	16 ± 0.6
Microshoot in vitro culture grown in PlantForm bioreactors	175.0	6 ± 0.4	76 ± 8.4	66 ± 7.3	14 * ± 0.6
	17.5	26 ± 1.8	41 * ± 4.5	48 * ± 5.3	17 ± 0.7
Schisantherin A and schisantherin B equimolar mixture	2.2	31 ± 1.6	38 ± 5.8	48 ± 5.3	8 ± 0.3
	0.2	55 ± 3.8	74 ± 8.1	31 ± 3.4	1 ± 0.04
Nordihydroguaiaretic acid (control)	30.2	23 ± 2.0	-	-	-
Ibuprofen (control)	2.1	-	23 ± 2.5	21 ± 2.0	-
Thioetheramide-PC (control)	73.6	-	-	-	91 ± 4.0

Asterisk (*) denotes statistical significance within the column for measured parameter between leaf and in vitro material according to Student *t*-test (at α < 0.05).

**Table 4:** Total phenolic content (estimated based on F-C assay) and antioxidant potential (estimated based on FRAP, DPPH, and CUPRAC assays) of *S. henryi* extracts from leaves and microshoots grown in PlantForm bioreactors and for equimolar schisantherin A and B mixture. Values are expressed in mmol Trolox equivalent per 100 g of plant material (DW) ± SD or schisantherin mixture.

Extracts	Assays			
	F-C	FRAP	DPPH	CUPRAC
Leaves of parent plant	9.0 * ± 0.4	2.4 * ± 0.04	5.9 * ± 1.1	6.7 * ± 0.2.
Microshoot in vitro culture grown in PlantForm bioreactors	37.0 * ± 0.2	13.5 * ± 0.2	19.5 * ± 2.1	25.9 * ± 0.7
Schisantherin A and schisantherin B equimolar mixture	24.5 ± 2.2	13.5 ± 0.5	101.3 ± 21.8	6.3 ± 2.1

Asterisk (*) denotes statistical significance within the column for measured parameter between leaf and in vitro material according to Student *t*-test (at α < 0.05).

**Table 5:** Antiproliferative activity [IC_50_ (µg/mL ± SD)] of *S. henryi* extracts from leaves and microshoots grown in PlantForm bioreactors and schisantherin B on cancer cells.

Extracts	Cancer Cells				Normal Cells
	HeLa	HT-29	MCF-7	Jurkat	HEK-293
Leaves of parent plant	65.2 * ± 3.3	43.57 * ± 2.1	73.63 * ± 3.2	85.48 * ± 2.7	>400
Microshoot in vitro culture grown in PlantForm bioreactors	78.57 * ± 2.5	57.64 * ± 1.9	81.53 * ± 2.7	94.21 * ± 3.2	>400
Schisantherin A and schisantherin B equimolar mixture	35.63 ± 1.2	24.35 ± 0.8	26.34 ± 1.3	44.62 ± 0.8	>400
Vinblastine sulfate (control)	2.2 ± 0.05	17.63 ± 0.5	-	0.1 ± 0.02	45.1 ± 0.5
Taxol (control)	-	-	0.06 ± 0.005	-	-

Asterisk (*) denotes statistical significance within the column for measured parameter between leaf and in vitro material according to Student *t*-test (at α < 0.05).

**Table 6:** Antimicrobial activity of *S. henryi* extracts from leaves and microshoots grown in PlantForm bioreactors against selected bacterial and fungi strains.

	Microorganisms	Leaves of Parent Plant			Microshoot In Vitro Culture Grown in PlantForm Bioreactors			Standard Drugs (Control)	
		MIC (mg/mL)	MBC or MFC (mg/mL)	MBC/MIC or MFC/MIC Ratio	MIC (mg/mL)	MBC or MFC (mg/mL)	MBC/MIC or MFC/MIC Ratio	MIC (mg/L)	MBC or MFC (mg/L)
								Vancomycin	
Gram-positive bacteria	*S. aureus* ATCC25923	1.25 *	10	8 *	5 *	10	2 *	0.98	0.98
	*S. aureus* ATCC43300	1.25 *	5	4 *	5 *	10	2 *	0.98	0.98
	*S. epidermidis* ATCC12228	1.25 *	2.5	2 *	5 *	5 *	1 *	0.98	0.98
								Ciplofloxacin	
Gram-negative bacteria	*E. coli* ATCC25922	10	10	1	10	10	1	0.015	0.08
	*P. aeruginosa* ATCC9027	10	10	1	10	10	1	0.488	0.98
	*H. pylori* ATCC 43504	0.625	0.625	1	0.625	0.625	1	0.98 ^†^	0.98 ^†^
								Nystatin	
Fungi	*C. albicans* ATCC 102231	5 *	10 *	2	10 *	20 *	2	0.24	0.48
	*C. parapsilosis* ATCC 22019	5 *	20	4 *	10 *	20	2 *	0.24	0.48
	*C. glabrata* ATCC 90030	10	10 *	1 *	10	20 *	2 *	0.48	0.48
	*A. niger* ATCC 16404	10	nd	nd	10	nd	nd	0.5 ^‡^	nd

nd—not detected; MIC—minimal inhibitory concentration; MBC—minimum bactericidal concentration; MFC—minimum fungicidal concentration; ^†^ ofloxacin; ^‡^ amphotericin B; asterisk (*) denotes statistical significance within the row for measured, corresponding parameters, between leaf and in vitro material according Student *t*-test (at α < 0.05).

## Data Availability

The authors confirm that the data supporting the findings of this study are available within the article and its Supplementary Materials.

## Supplementary Materials

The following supporting information can be downloaded at: https://www.mdpi.com/article/10.3390/ph17040442/s1, Table S1. Chemical structures of detected lignans in extracts in biomass of *S. henryi*. Figure S1. Exemplary TIC (total ion current) chromatogram of acquired MRM (multiple reaction monitoring) of lignan authentic standard mixture: 1—wulignan A1; 2—rubrisandrin A; 3—rubriflorin A; 4—schisandrin; 5—gomisin D; 6—gomisin J; 7—gomisin A; 8—gomisin G; 9—licarin B; 10—epigomisin O; 11—gomisin O; 12—schisantherin A; 13—schisantherin B; 14—licarin A; 15—schisanhenol; 16—deoxyschisandrin; 17—fragransin A; 18—pregomisin; 19—gomisin N; 20—6-O-benzylgomisin O; 21—schisandrin C.
