# Supplementary material for: Comparative Assessment of Lignan Profiling and Biological Activities of Schisandra henryi Leaf and In Vitro PlantForm Bioreactor-Grown Culture Extracts
Source: Pharmaceuticals (Basel). 2024 Mar 29;17(4):442. doi: 10.3390/ph17040442 (PMC11053505; doi:10.3390/ph17040442)
Supplement: Supplementary file 1 [file pharmaceuticals-17-00442-s001.zip › pharmaceuticals-2903952-supplementary.pdf]

Supplementary data

Comparative assessment of lignan profiling and biological activities of *Schisandra henryi* leaf and in vitro PlantForm bioreactor grown culture extracts

Karolina Jafernik [1], Paweł Kubica [1], Michał Dziurka [2], Łukasz Kulinowski [3], Izabela Korona-Głowniak [4], Hosam O. Elansary [5], Piotr Waligórski [2], Krystyna Skalicka-Woźniak [3] and Agnieszka Szopa [1,*]

[1] Department of Pharmaceutical Botany, Medical College, Jagiellonian University, Medyczna 9 str, 30-688 Kraków, Poland; karolina.jafernik@doctoral.uj.edu.pl (K.J.), p.kubica@uj.edu.pl (P.K.),a.szopa@uj.edu.pl (A.S.)

[2] Polish Academy of Sciences, The Franciszek Górski Institute of Plant Physiology, Niezapominajek 21 str, 30-239 Kraków, Poland; michal.dziurka@gmail.com (M.D.) p.waligorski@ifr-pan.edu.pl (P.W.)

[3] Department of Natural Products Chemistry, Medical University of Lublin, Chodźki 1 str, 20-093 Lublin, Poland; lukasz.kulinowski@umlub.pl (Ł.K), kskalicka@pharmacognosy.org (K.S.-W.)

[4] Department of Pharmaceutical Microbiology, Medical University of Lublin, 1 Chodźki str., 20-093 Lublin, Poland; iza.glowniak@umlub.pl (IKG)

[5] Department of Plant Production, College of Food & Agriculture Sciences, King Saud University, P.O. Box 2460 str, Riyadh 11451, Saudi Arabia; helansary@ksu.edu.sa (H.O.E.)

* Correspondence: a.szopa@uj.edu.pl (A.S.)

Table S1. Chemical structures of detected lignans in extracts in biomass of *S. henryi*

Compound	Chemical structure	Transition	Acquisition channel
**Wulignan A1**	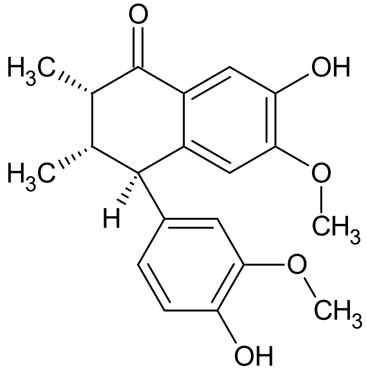	343.3 -> 117.0	MRM
**Rubrisandrin A**	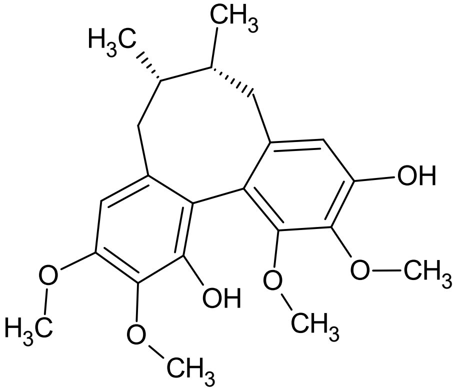	369.3 -> 351.1	MRM

**Rubriflorin A**	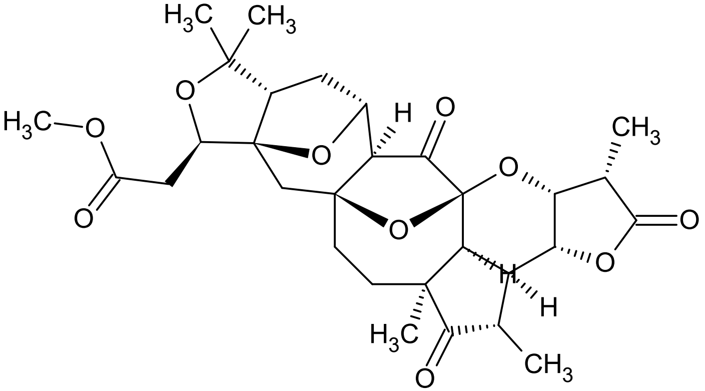	557.4 -> 173.0	MRM
**Schisandrin**	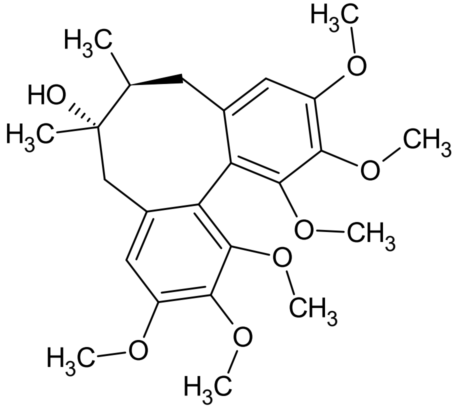	415.3 -> 359.2	MRM

**Gomisin D**	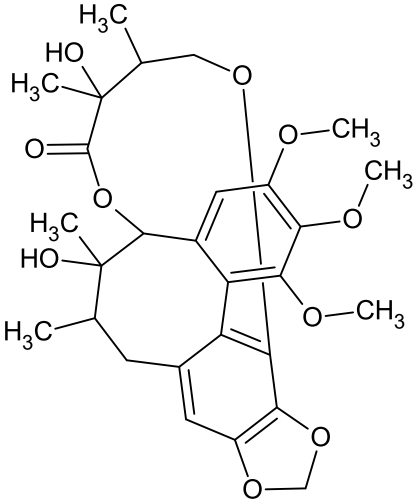	401.3 -> 168.0	MRM
**Gomisin J**	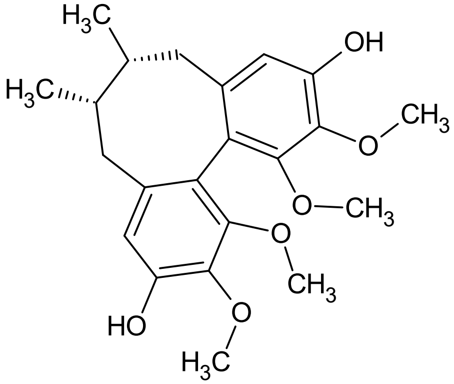	389.3 -> 117.0	MRM

**Gomisin A**	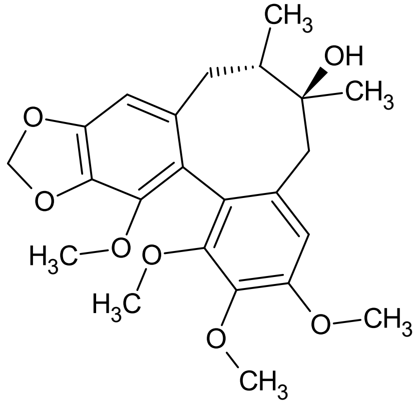	399.3 -> 368.2	MRM
**Gomisin G**	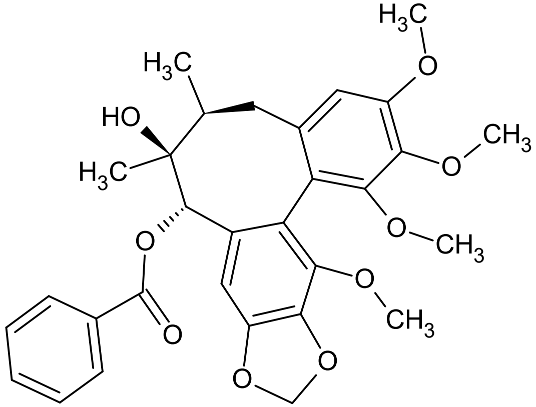	415.3 -> 371.1	MRM
**Licarin B**	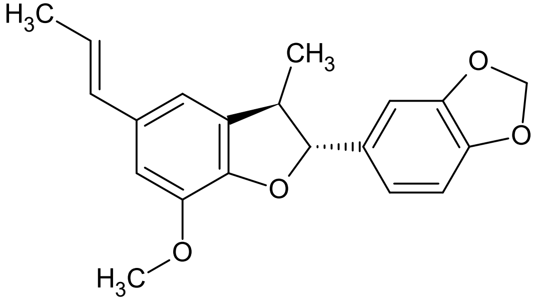	399.3 -> 368.2	MRM

**Epigomisin O**	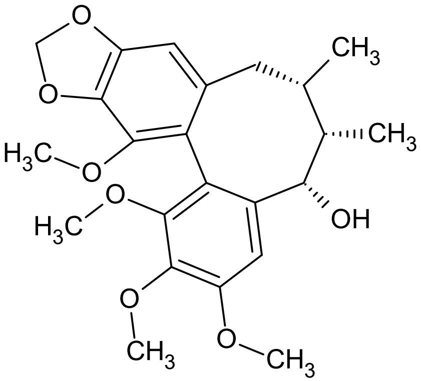	399.3 -> 368.2	MRM
**Gomisin O**	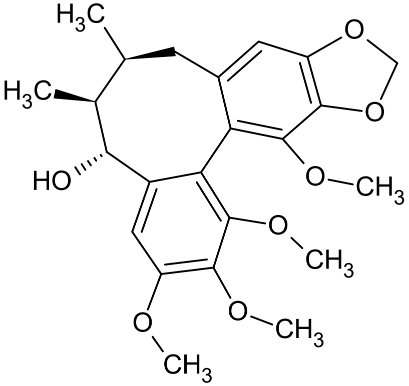	325.2 -> 152.0	MRM

**Schisantherin A**	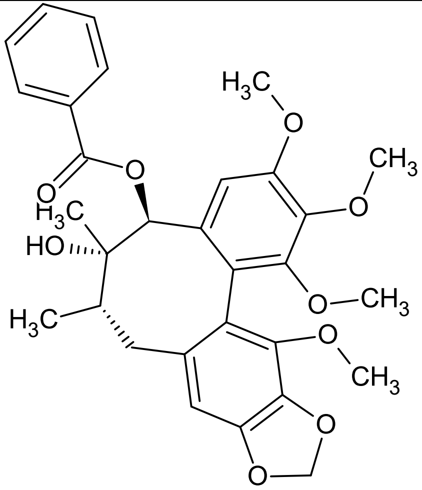	415.3 -> 371.1	MRM
**Schisantherin B**	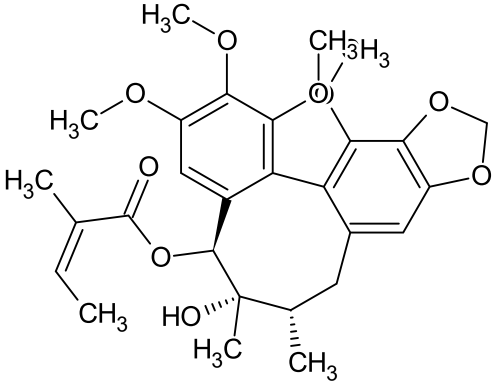	415.3 -> 371.1	MRM
**Licarin A**	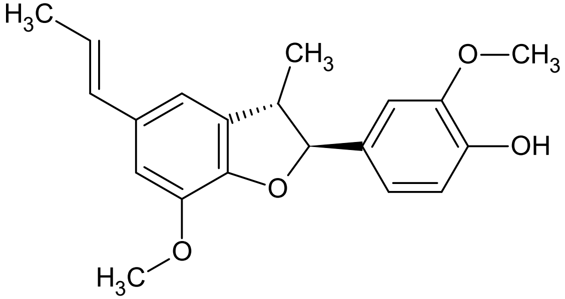	327.3 -> 105.1	MRM

**Schisanhenol**	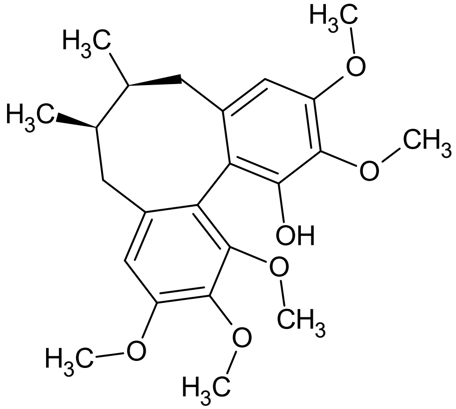	403.3 -> 231.0	MRM
**Deoxyschisandrin**	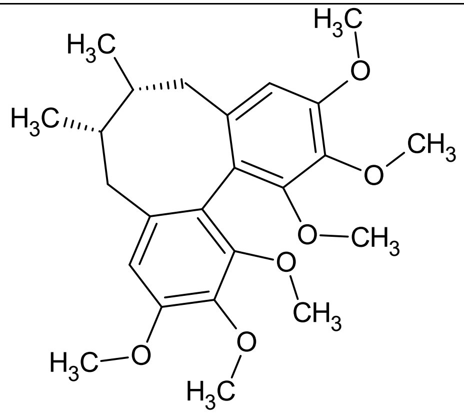	417.3 -> 316.2	MRM
**Gomisin N**	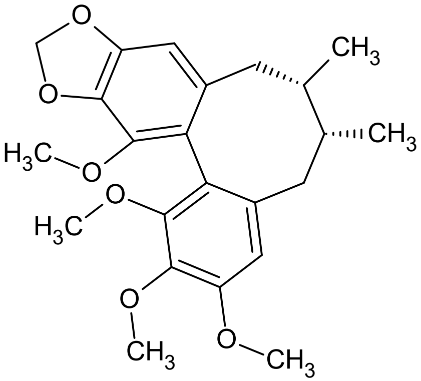	401.3 -> 168.0	MRM

**6-O-benzylgomisin O**	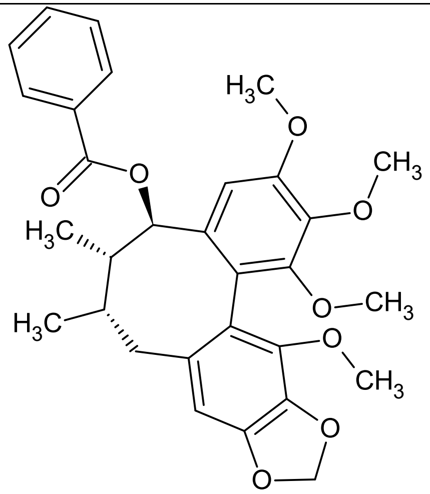	399.3 -> 368.2	MRM

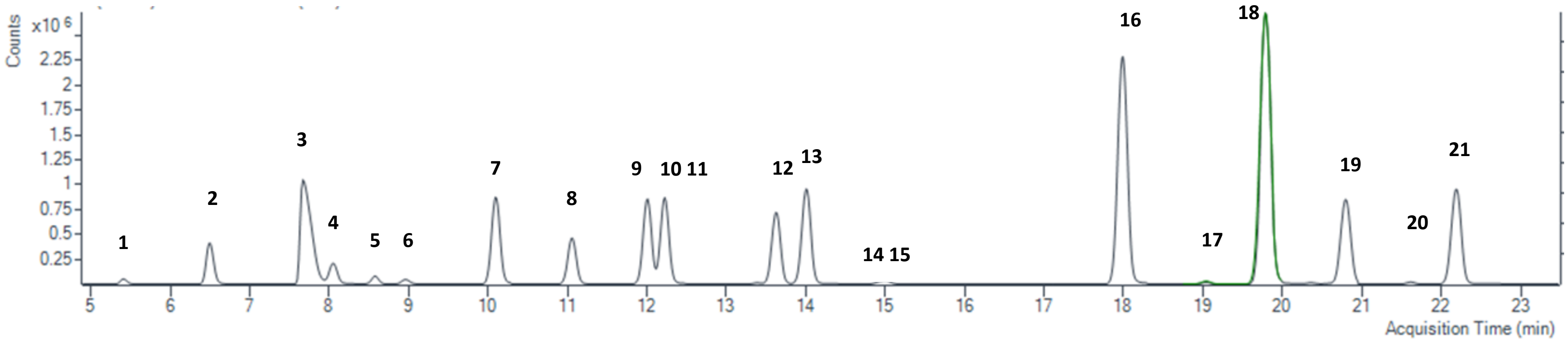

Figure S1. Exemplary TIC (total ion current) chromatogram of acquired MRM (multiple reaction monitoring) of lignan authentic standard mixture: 1 - wulignan A1; 2 - rubrisandrin A; 3 - rubriflorin A; 4 – schisandrin; 5 - gomisin D; 6 - gomisin J; 7- gomisin A; 8 - gomisin G; 9 - licarin B; 10 - epigomisin O; 11 - gomisin O; 12 - schisantherin A; 13 - schisantherin B; 14 - licarin A; 15 – schisanhenol; 16 – deoxyschisandrin; 17 - fragransin A; 18 – pregomisin; 19 - gomisin N; 20 - 6-O-benzylgomisin O; 21 - schisandrin C.
